# Remedies and Their Uses

**Published:** 1907-03-16

**Authors:** 


### Remedies and their Uses.

### Peroxides and Nascent Oxygen: Powerful Deoxidisers and Deodorants.

Peroxide of hydrogen ($H_2O_2$) has long been familiarly known, not only to the chemist, but to the scientific and medical world in general. Its remarkable properties and constitution give it a certain prominence, and as its oxidising powers are readily appreciated by anyone with the slightest knowledge of elementary chemistry, it seems curious that it should not have been more widely used. The official preparation is the *liquor hydrogenii peroxidi.* Its strength is generally spoken of as so many "volumes," signifying the number of volumes of oxygen which can be extracted from it. The official solution gives off between nine and eleven times its own volume of oxygen. This solution is said to equal in disinfectant power 1 in 1,000 perchloride of mercury. A little acid is added to this solution to prevent decomposition, which is somewhat prone to occur, and the presence of this acid in excess may give rise to unpleasant results. Several other preparations, improvements on the pharmacopœial liquor, are on the market, of which may first be mentioned

*Perhydrol* (Merck).

This is a 30 per cent. solution by weight, and yields 100 volumes of oxygen. It is chemically pure, and free from any trace of acid, though the substance itself causes slight reddening of litmus. It keeps well, and is sent out in specially prepared bottles containing 50 and 200 grains respectively (about $1\frac{3}{4}$ and 7 fluid ounces). It is non-toxic and non-irritant, and is a powerful deodorant.

Internally it has been used for septic and diphtheritic affections of the throat, one drachm of perhydrol being mixed with half an ounce of glycerine, and 9 ounces of distilled water. One drachm doses of this mixture may be taken every quarter of an hour. It may also be given in the intestinal diseases of children, especially in acute diarrhœa; enemata containing 5 per cent. of perhydrol in distilled water may be injected and retained several times daily. In atonic dyspepsia and constipation perhydrol acts as an intestinal stimulant; in gastritis and in the vomiting of alcoholics it may be given in doses of 10 minims, diluted up to 3 or 4 ounces with distilled water and syrup of orange. It should not be given in gastric ulcer, however, as it is apt to cause an increase in the pain.

Externally a solution of perhydrol may be used for washing out discharging sinuses and cavities, and for loosening dressings which have become stuck to the raw surfaces of wounds, a 2.5 per cent. solution being employed. In inoperable cancer it forms a valuable disinfectant. It is also markedly hæmostatic, and may be used for an indefinite time, as it can produce no toxic symptoms. Favus, a very resistant disease, has been cured by 5 per cent. solution applied on compresses and changed every two hours. Syphilitic and other ulcers and gangrenous conditions of the skin are much improved by perhydrol, which in some cases has been applied pure. It is valuable in stomatitis, a very dilute solution (1 per cent.) being used as a mouth wash.

In cystitis, perhydrol 1 in 300 up to 1-100 may be used to wash out the bladder, and in gonorrhœa valuable results have been obtained by intraurethral injections of 1 in 100 to 1 in 200 perhydrol, combined with .1 in 100 to .1 in 400 silver nitrate. In conjunctivitis and in various forms of corneal ulcer, solutions varying from .5 per cent. to 3 per cent. have been used as douches, on compresses, and for painting the lids. Perhydrol, owing to its neutral reaction, is specially useful in otitis media. Solutions of the ordinary strength may be employed, and, if necessary, gradually increased in concentration till 10 per cent. or even 15 per cent. is employed. Its hæmostatic action renders it very useful as a dental antiseptic. In gynæcology it may be used in all suppurative and ulcerative processes of the vaginal mucous membrane, in cervical erosions, and as an intra-uterine douche in septic endometritis.

Other preparations which depend for their activity on the oxidising properties of nascent oxygen are the peroxides of magnesium and zinc. Merck has preparations on the market corresponding to perhydrol, which are similarly named.

*Magnesium Perhydrol* (Merck)

is a white powder, insoluble in water, containing 15-20 per cent. $MgO_2$, the rest being the ordinary oxide MgO. It is intended for internal use in gastro-intestinal diseases. Flatulence, vomiting, and nausea may be treated by cachets containing 4 to 8 grains of this preparation. In diarrhœa, especially apparently in phthisis, it has also been advantageously employed. In this case keratin-coated capsules should be used, in order to avoid gastric digestion. It may also be given three or four times daily, half an hour before or after meals, in doses of half to one dram suspended in water. In this form the monoxide may have a slight laxative action.

*Zinc Perhydrol* (Merck)

is also a white powder, insoluble in water. It contains 50 per cent. $ZnO_2$ and 50 per cent. ZnO. In the presence of acids nascent oxygen is evolved. Sodium peroxide, which was originally suggested as an antiseptic, has the disadvantage of forming the caustic NaOH on decomposition; the corresponding zinc compound is harmless. It should, if ordered as an ointment, be combined with a vaseline or paraffin base (10 per cent. strength), as with a fatty base irritating zinc compounds may be formed.

Preparations of these peroxides have also been introduced under the trade-names of *Hopogan* ($MgO_2$) and *Ectogan* ($ZnO_2$). Gilbert notes that in twenty cases out of twenty-two good results were obtained in the following gastric disorders: constant furred tongue and fœtid breath, excessive eructations, vomiting, and epigastric discomfort with cardiac oppression after meals. In a day or two improvement may be noted, but the treatment should be continued for several days after the last symptoms have disappeared.

